# The DPP-4 inhibitor sitagliptin attenuates the progress of atherosclerosis in apolipoprotein-E-knockout mice via AMPK- and MAPK-dependent mechanisms

**DOI:** 10.1186/1475-2840-13-32

**Published:** 2014-02-04

**Authors:** Yanmei Zeng, Chenzhong Li, Meiping Guan, Zongji Zheng, Jingjing Li, Wenwei Xu, Ling Wang, Feiying He, Yaoming Xue

**Affiliations:** 1 Department of Endocrinology and Metabolism, Nanfang Hospital, Southern Medical University, Guangzhou 510150, China

**Keywords:** Sitagliptin, Atherosclerosis, Inflammation, AMPK signaling pathway MAPK signaling pathway, Leukocyte–endothelial cell interaction

## Abstract

**Background:**

The dipeptidyl peptidase-4 inhibitor sitagliptin, a new anti-diabetic medicine, is effective in treating type 2 diabetes mellitus by increasing the activation and duration of action of glucagon-like peptide-1. Since atherosclerosis is the main pathological feature of diabetic cardiovascular complications, it is important to investigate the anti-atherosclerotic effect of sitagliptin and explore the relevant mechanisms.

**Methods:**

Male apolipoprotein-E-knockout mice were randomly divided into two groups and fed either high-fat diet (HFD) or HFD plus sitagliptin at a concentration of 0.3% for 16 weeks. Body weight, food intake, blood glucose, serum lipids and adhesion molecules were measured. The atherosclerotic plaque area and its histological composition were analyzed using Sudan staining and immunohistochemistry. The expression of inflammatory cytokines (monocyte chemoattractant protein (MCP)-1 and interleukin (IL)-6) and the activation of AMP-activated protein kinase (AMPK) and mitogen-activated protein kinase (MAPK) in the aortas were determined using quantitative polymerase chain reaction and western blot, respectively.

**Results:**

Mice treated with sitagliptin developed fewer atherosclerotic plaques than the control group (7.64 ± 1.98% vs 12.91 ± 1.15%, p < 0.001), particularly in the aortic arch and abdominal aorta, where plaques were decreased 1.92- and 2.74-fold, respectively (p < 0.05 and p < 0.01). Sitagliptin significantly reduced the content of collagen fiber in plaques 1.2-fold (p < 0.05). Moreover, sitagliptin significantly reduced the expression of monocyte chemoattractant protein-1 and interleukin-6 in the aorta (p < 0.01 and p < 0.05), as well as the serum levels of soluble vascular cell adhesion molecule-1 and P-selectin (both p < 0.05). In addition, Sitagliptin induced phosphorylation of AMPK and Akt (p < 0.05 and p < 0.01), while suppressed phosphorylation of p38 and extracellular signal-regulated kinase (Erk) 1/2 (p < 0.05 and p < 0.01) in aortas.

**Conclusions:**

Our present study indicates that sitagliptin can reduce the area of the atherosclerotic lesion, possibly by regulating the AMPK and MAPK pathways and then reducing leukocyte –endothelial cell interaction and inflammation reactions. These actions are independent of weight loss and glucose-reducing effects.

## Background

Atherosclerosis, one of the most important manifestations and main pathological features of diabetic vascular complications, is a chronic inflammatory response in arteries that is caused by the recruitment of blood monocytes, deposition of lipids, and formation of macrophage foam cells. Atherosclerosis remains the leading cause of morbidity and mortality in patients with type 2 diabetes mellitus [1].

DPP-4 inhibitors are a new class of anti-diabetic drugs that improve glucose metabolism by raising the active concentration and duration of action of glucagon-like peptide (GLP)-1 [2-5]. Sitagliptin, as one of the DPP-4 inhibitors, has been reported to play a protective role in the cardiovascular disease included atherosclerosis [6-11]. But the mechanisms through which sitagliptin attenuate the progress of atherosclerosis are complex and still not completely understood. It was previously reported that most of the anti-atherosclerotic effects of GLP-1 and DPP-4 inhibitors may be mediated through the activation of intracellular cyclic AMP (cAMP) and protein kinase A (PKA) signaling [6,12]. However, other studies have indicated that DPP-4 inhibitors might protect against endothelial inflammation and increase nitric oxide (NO) through other mechanisms, independent of the cAMP/PKA or phosphatidylinositide 3-kinase (PI3K)/AKT pathways [13,14]. Recently researches confirmed that Sitagliptin and exendin-4 can not only activate the phosphorylation of AMPK but also inhibit the activation of MAPK including p38 and ERK [15-18]. Activation of AMP-activated protein kinase (AMPK), an energy sensor ubiquitously expressed in vascular cells, has been reported to possess anti-atherosclerotic effects [19-21] by upregulating the Akt/endothelial NO synthase (eNOS)/NO signaling pathway, leading to the suppression of p38-mediated nuclear factor-κB activation and, consequently, suppression of downstream inflammatory responses [21-23]. And suppression of mitogen-activated protein kinase (MAPK) also has been reported to have beneficial effects in atherosclerosis through inhibiting adhesion molecules and anti-inflammation effects, as well as increase the stability of the carotid plaques [24-29].

Thus, based on the indication of above researches and the proposed effects of sitagliptin, we hypothesized that sitagliptin can inhibit the progression of atherosclerosis possibly by activating the AMPK and suppressing the MAPK, leading to decreases in adhesion molecules and inflammatory cytokines. To test this hypothesis, we conducted this comprehensive study to evaluate the anti-atherosclerotic effect of sitagliptin and explore the underlying mechanisms in ApoE-/- mice.

## Methods

### Animals and diets

ApoE-/- mice with the C57BL/6 genetic background, provided by Joslin Diabetes Center (Boston, MA, USA), were bred in a pathogen-free environment with a 12 h light/dark cycle and free access to food and water. We performed the experimental research on animals following internationally recognized guidelines with the approval of an appropriate ethics committee. All experiments were performed in the experimental animal center of Southern Medical University, Guangzhou, China (certificate number: SCXK2011-0015) according to institutional and government guidelines and approved by the local council of ethics. At the age of 8 weeks, 24 male ApoE-/- mice were randomly divided into two groups. The control group was fed a high-fat diet (GDLMC, Guangzhou, China) containing 21.8% fat, providing 42% energy and 1.25% cholesterol for 16 weeks, while the experimental group was fed a high-fat diet mixed with sitagliptin (Merck & Company, Guangzhou, China) at a final concentration of 0.3% (corresponding to 200 mg/kg/day) for the same time.

### Metabolic profile analysis

The body weight and food intake of animals were recorded weekly. An intraperitoneal glucose tolerance test, with injection of 20% glucose at a dose of 2 g/kg, was performed at week 14 after 8 h fasting using tail vein blood with the One Touch Ultra (Lifescan; Johnson & Johnson, USA) at 0, 15, 30, 60, and 120 min. At the end of the study, all of the mice were fasted for 8 hours, and blood samples were then collected from the orbital sinus after inhalation of CO2. Serum centrifuged from the blood samples was used to measure levels of plasma lipids such as triglycerides (TG), total cholesterol (TC), high-density lipoprotein cholesterol(HDL), low-density lipoprotein cholesterol (LDL), and very low-density lipoprotein cholesterol (VLDL) using an automatic biochemical analyzer (Dimension, USA). In addition, serum levels of the soluble adhesion molecules vascular cell adhesion molecule (VCAM)-1 and P-selectin were determined by enzyme-linked immunosorbent assay (R&D Systems, UK), performed according to the manufacturer’s instructions. Aortas and other tissues were also collected and quickly frozen in liquid nitrogen, and then stored at -80°C for later analysis.

### Quantification of atherosclerotic lesion area

After removing the adventitial fatty tissue, aortas were opened longitudinally from the aortic root to the renal artery, and fixed in 10% formalin for 36 h. And then the fixed aortas were stained with Sudan IV for 10 min, differentiated in 70% alcohol for 15 min, and washed in water for 30 min. To quantify area of the atherosclerotic lesion, the stained aortas were photographed using a digital camera connected to a dissection microscope, and then evaluated it as the ratio of the positive area to the total aortic area by Image-Pro Plus 6.0.

### Immunohistochemistry measurements of atherosclerotic plaques

To analyze the histological composition of atherosclerotic plaques, aortas fixed in 10% formalin after 24 h were paraffin embedded and cross-sectioned. Masson’s trichrome stain kit (Maiwei, Xiamen, China) was used to assess the collagen fiber content of the lesions, and immunohistochemistry was used to qualify the levels of vascular smooth muscle cells and macrophage cells. Immunohistochemistry was performed as follows: cross-sections of aortas were incubated with goat anti-macrophage-2 antigen mouse macrophage or anti-alpha-SMA polyclonal antibody, and then incubated with a biotinylated secondary antibody; and finally counterstained with hematoxylin (Bioss, Beijing, China). All cross-sections were analyzed under an upright microscope (Nikon, Tokyo, Japan). The expression levels of macrophage and smooth muscle cells, as well as of collagen fiber, were evaluated using Image-Pro Plus 6.0.

### Determination of inflammatory cytokine mRNA levels through RT-PCR

SYBR green quantitative real-time polymerase chain reaction (RT-PCR) was used to detect the mRNA levels of DPP-4 and GLP-1 receptor (GLP-1R), as well as of the inflammatory cytokines monocyte chemoattractant protein (MCP)-1 and interleukin (IL)-6. RNA extracted from the aortic tissue of mice with E.Z.N.A Total RNA Kit II (Omega, USA) was reversed to cDNA using the PrimeScript RT reagent Kit (Takara Biotechnology, Japan) with the following profile conditions: 37°C for 15 min, 85°C for 5 seconds, and 4°C for ever. Quantitative real-time PCR was performed with ABI 7500 (ABI, USA) using SYBR Premix Ex Taq (Takara Biotechnology, Japan) as follows: one cycle at 95°C for 30 min; 40 cycles at 95°C for 5 seconds and 60°C for 34 seconds; and one cycle at 95°C for 15 seconds, 60°C for 1 minute, and 95°C for 15 seconds. The relative quantification values for these gene expressions were calculated by ΔΔCT methods and corrected using a housekeeping gene. The primers used were as follows: glyceraldehyde 3-phosphate dehydrogenase(GAPDH)forward 5'-GTGAAGCAGGCATCTGAGGG-3' and reverse 5'-CGAAGGTG GAAGAGTGGGAGT-3'; DPP-4 forward 5'-GTCTAAGCGAGGGGAGAGAAAC-3' and reverse 5'-CAAGGCGGAGAAAGAAAGTG-3'; GLP-1R forward 5'-TGACCGACTGTTTGTTCTCTTG-3' and reverse 5'-CCAACTTATGACCTTCTGGTGAC-3'; MCP-1 forward 5'-GCAGCAGGTGTCCCAAAGAA-3' and reverse 5'-ATTTACGGGTCAACTTCACATTCAA-3'; and IL-6 forward 5'-AAAGCTGCGCAGAATGAGATG-3' and reverse 5'-AAAGCTGCGCAGAATGAGATG-3'.

### Phosphorylation of AMPK and MAPK via western blot analysis

SDS-PAGE immunoblotting was used to quantified the phosphorylation of AMPK and MAPK signaling molecules, including Akt, extracellular signal-regulated kinase (ERK)1/2, and p38. Aortic tissues were ground on ice with 250 μl RIPA buffer per 20 mg, and then centrifuged at 12,000 g for 20 min to obtain the aortic protein. The protein abundance was detected with antibodies against phospho-AMPK, AMPK, phospho-Akt, Akt, phospho-p38, p38 phospho-ERK1/2, and ERK1/2 (Bioworld, USA). And then anti-rabbit fluorescence secondary antibody was used for chemiluminescene detection. Images were obtained using infrared scanning (Odyssey, USA) and quantified using GelPro32, and calculated by the ratio of phosphorylation to the total protein level.

### Statistical analysis

All data are expressed as mean ± SD. Comparisons of means between two groups were analyzed using an unpaired Student’s t test. p values <0.05 were considered significant. All analyses were performed using SPSS version 13.0 for Windows.

## Results

### Animal data and metabolic profile

**Figure 1:**
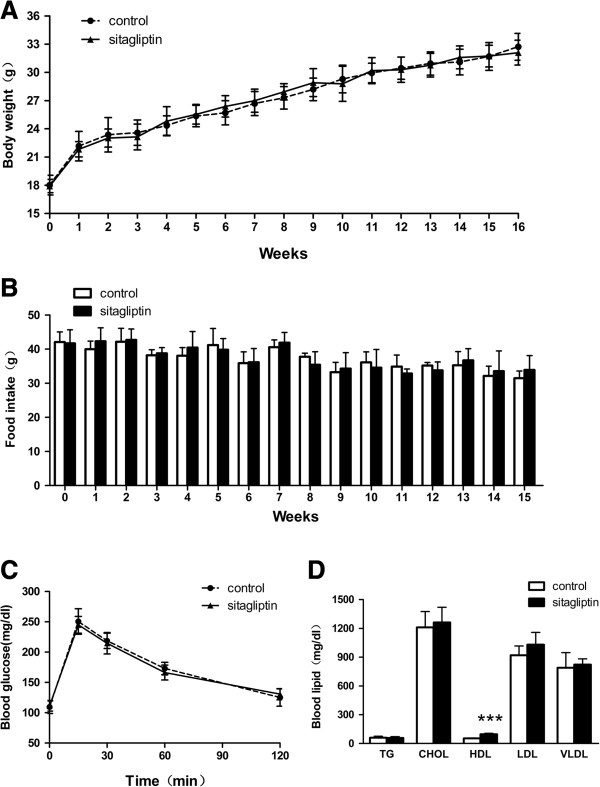
**Characteristics of control mice and those fed sitagliptin.** There were no significant differences in body weight **(A)**, food intake **(B)**, blood glucose **(C)**, or blood lipids **(D)** between the two groups, except for a significantly increased HDL cholesterol level in mice in the sitagliptin group. Data are mean ± SD, n = 12 per group.

Table 1 shows the data of body weights, food intake, and blood glucose and lipid levels of the animals. Statistical analysis showed that there were no significant differences in body weight, food intake, or blood glucose levels between the two groups during the period of the experiment (Figure 1A–C). Moreover, our data indicated that sitagliptin can significantly increase the level of HDL (52.78 ± 5.25 vs 97.76 ± 8.56, p < 0.001), and tends to increase the LDL cholestrerol levels in ApoE-/- mice, whereas with no effect on TC, CHOL or VLDL compared with the control group (Figure 1D).

**Table 1:** Data on body weight, food intake, fasting blood glucose, and blood lipid profile in both groups

	**Control**	**Sitagliptin**	**p value**
Daily food intake (g/w)	37.13 ± 3.24	37.41 ± 3.57	0.82 (NS)
Body weight pretreatment (g)	18.01 ± 1.04	17.91 ± 0.71	0.79 (NS)
Body weight (g)	32.73 ± 1.43	32.1 ± 1.32	0.28 (NS)
Fasting blood glucose (mg/dl)	109.58 ± 10.65	111.09 ± 8.30	0.76 (NS)
Total cholesterol (mg/dl)	1211.02 ± 163.79	1260.41 ± 160.12	0.55 (NS)
Triglycerides (mg/dl)	60.73 ± 15.62	58.19 ± 12.94	0.73 (NS)
HDL cholesterol (mg/dl)	52.78 ± 5.25	97.76 ± 8.56	<0.001
LDL cholesterol (mg/dl)	919.61 ± 96.49	1030.10 ± 128.70	0.07 (NS)
VLDL cholesterol (mg/dl)	789.24 ± 157.62	821.99 ± 59.54	0.60 (NS)

HDL: high-density lipoprotein; LDL: low-density lipoprotein; TG: triglyceride; VLDL: very low-density lipoprotein. Values are expressed as mean ± SD, n = 8–12 per group.

### Sitagliptin reduced atherosclerotic lesion formation in ApoE-/- mice

**Figure 2:**
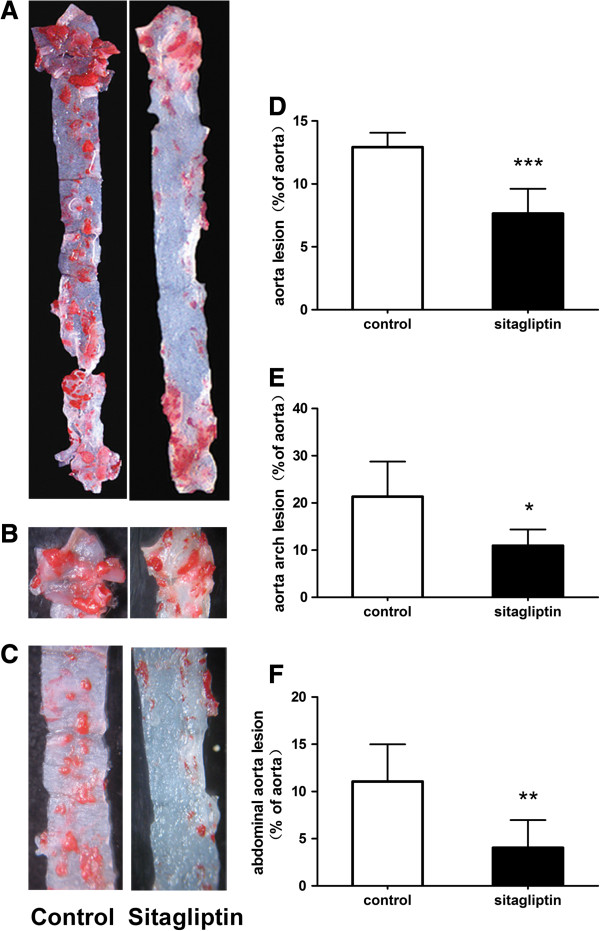
**Atherosclerotic lesion formation in aortic sections from ApoE-/- mice.** Sitagliptin reduced atherosclerotic lesion formation in ApoE-/- mice. **(A)** Aortic en face flat from the aortic root to the renal artery stained with Sudan IV. **(B)** En face flat of the aortic arch. **(C)** Aortic en face flat of the abdominal aorta. **(D)** Relative atherosclerotic lesion area of the atherosclerotic lesion to the whole aorta in the two groups. **(E)** Relative atherosclerotic lesion area in the aortic arch. **(F)** Relative atherosclerotic lesion area in the abdominal aorta. Data are mean ± SD, n = 6. *p < 0.05, **p < 0.01 versus control.

The Sudan IV staining results show the atherosclerotic plaques in entire aorta, aortic arch and abdominal aorta (Figure 2A–C). In ApoE-/- mice, the sitagliptin group showed fewer atherosclerotic plaques than in controls (7.64 ± 1.98% [range 4.62–10.13%] vs 12.91 ± 1.15% [range 11.55–14.37%], p < 0.001; Figure 2D). Compared with control mice, atherosclerotic plaque areas decreased respectively 1.92- and 2.74-fold in the aortic root and abdominal aorta of mice fed sitagliptin (p = 0.011 and p = 0.006; Figure 2E, F). Our data show that sitagliptin can inhibit the formation of atherosclerotic areas in entire aorta, aortic root and abdominal aorta of ApoE-/- mice.

### Sitagliptin altered the content of macrophages and smooth muscle cells, as well as collagen fiber, in atherosclerotic lesions

**Figure 3:**
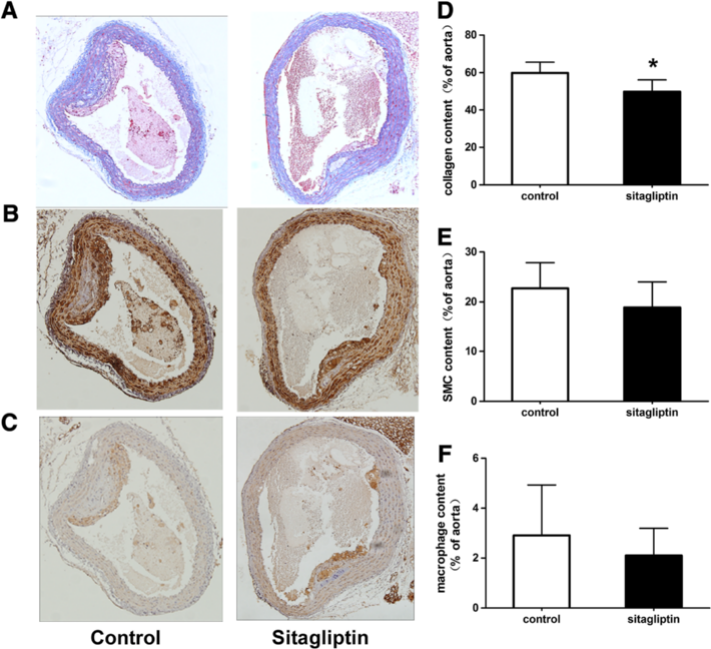
**Effects of sitagliptin on collagen fibers, macrophages, and smooth muscle cells in atherosclerotic lesions.** Cross-sections of the aortic root were cut into slices and stained with Masson **(A)**, anti-smooth muscle cell **(B)**, and anti-macrophage antibody **(C)**. The ratios occupied by collagen fibers **(D)**, smooth muscle cells **(E)**, and macrophages **(F)** in the aortic plaque were also evaluated. Values are mean ± SD; n = 6 per group; *p < 0.05.

Aorta cross-section immunochemistry results showed that there was significantly less collagen fiber in aortic plaques after intervention with sitagliptin in ApoE-/- mice (49.86 ± 6.26 um2 vs 59.83 ± 5.82 um2; p < 0.05; Figure 3A, D). The area occupied by vascular smooth muscle cells and macrophages tended to be decreased in the sitagliptin group compared to controls, albeit with no significant difference in the statistical analysis (Figure 3B, C, E, F). Alterations in the composition of the atherosclerotic area indicated that sitagliptin can change the stability of the atherosclerotic plaque and attenuate the progress of atherosclerosis.

### Effect of sitagliptin on mRNA expression levels of DPP-4, GLP-1R, and inflammatory cytokines in aortic tissues

**Figure 4:**
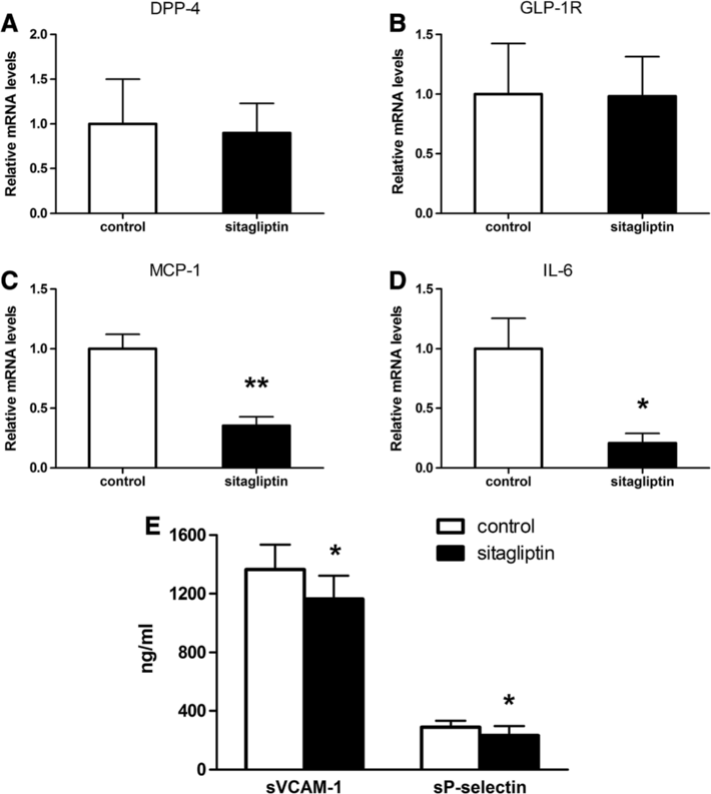
**Sitagliptin decreases the expression of proinflammatory cytokines and serum soluble adhesive molecules in ApoE-/- mice.** Messenger (m)ribonu-cleic acid RNA (mRNA) expression levels, relative to those of glyceraldehyde 3-phosphate dehydrogenase (GAPDH), of dipeptidyl peptidase (DPP)-4 **(A)**, glucagon-like peptide (GLP)-1 receptor **(B)**, monocyte chemoattractant protein (MCP)-1 **(C)**, and interleukin (IL)-6 **(D)**, as measured by quantitative real-time reverse transcriptase polymerase chain reaction in aortas (n = 6 each). Values represent the fold of the mean value measured in controls and are shown as mean ± SD; *p < 0.05, **p < 0.01. **(E)** Serum soluble vascular cell adhesion molecule (VCAM)-1 and P-selectin concentrations, as detected by ELISA (n = 10). Values are mean ± SD; *p < 0.05.

We confirmed that DPP-4 and GLP-1R mRNA expression levels in aortic tissues had no significantly differences between the control and sitagliptin groups (p > 0.05; Figure 4A, B). However, sitagliptin did significantly reduce the expression of MCP-1 and IL-6 (p = 0.001 and p = 0.033; Figure 4C, D), which mediate inflammation in the aorta. These data show that sitagliptin can inhibit inflammation in aortic tissues, but with no effect on the expression of DPP-4 and GLP-1R in the aortas.

### Sitagliptin reduced adhesion molecule expression in serum

The expression of cell adhesion molecules can mediate leukocyte rolling and leukocyte–endothelial cell interactions during inflammation. Compared to control mice, the serum levels of soluble VCAM-1 and P-selectin decreased significantly in the sitagliptin group (VCAM-1: 1163.16 ± 159.62 ng/ml vs 1365.18 ± 170.26 ng/ml, p < 0.05; P-selectin: 232.71 ± 64.29 ng/ml vs 288 ± 44.46 ng/ml, p < 0.05; Figure 4E). This demonstrates that sitagliptin can inhibit the expression of adhesion molecules in serum. Such molecules play an important role in the early formation of atherosclerosis by mediating the adhesive attraction of monocytes and endothelial cells.

### Effect of sitagliptin on the phosphorylation of the AMPK and MAPK signaling pathways

**Figure 5:**
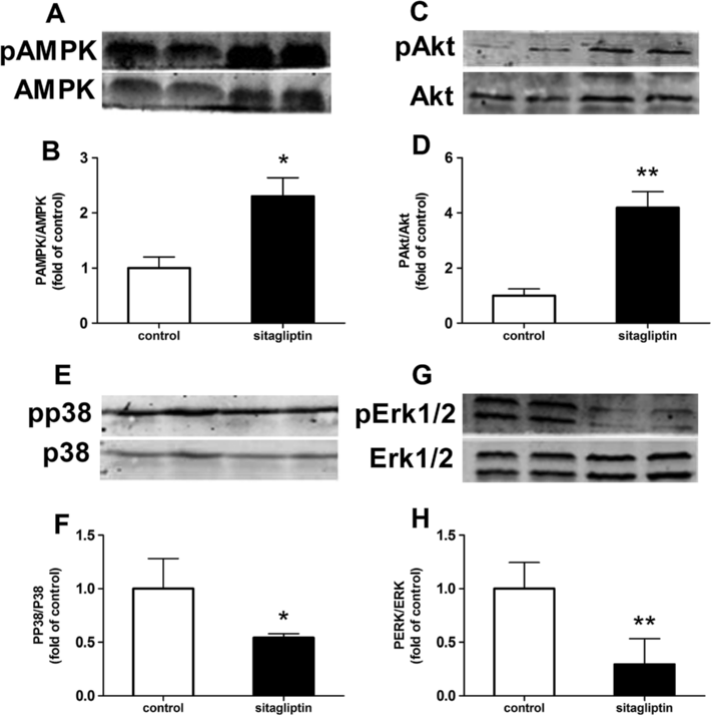
**Effects of sitagliptin on AMPK- and AMPK-mediated Akt activation and MAPK phosphorylation in the aorta.** Phosphorylative and total protein expression of AMPK **(A)**, Akt **(C)**, p38 **(E)** and ERK1/2 **(G)** in the aortic tissue of ApoE-/- mice. Quantitative analysis of pAMPK/AMPK **(B)**, pAkt/Akt **(D)**, pp38/p38 **(F)** and pERK1/2/ERK1/2 **(H)** in aortic tissue (n = 4 each). Values are mean ± SD; *p < 0.05, **p < 0.01.

Compared to controls, phosphorylation of AMPK and its downstream signaling molecule Akt increased in the aortic tissue of the sitagliptin group(p < 0.05 and p < 0.01), while the phosphorylation of p38 and ERK1/2 decreased (p < 0.05 and p < 0.01) (Figure 5). These data show that sitagliptin can activate the AMPK signaling pathway and inhibit the MAPK signaling pathway, which have already been shown to play a key role in anti-inflammation and anti-atherosclerotic effects.

## Discussion

The main findings of this study lie in the effects of sitagliptin in reducing the formation of atherosclerotic lesion area in the aortic root and abdominal aorta, and changing the histological composition of atherosclerotic plaques by reducing the content of collagen fiber and tending to reduce smooth muscle cells and macrophages in the aorta, although with no statistical significance. These results are consistent with previous researches on the anti-atherosclerotic effects of DPP-4 inhibitors in animals [6-11,30-34]. It has been shown that sitagliptin is cardioprotective in the diabetic Akita mouse even at low doses [32]. A pooled analysis of 25 randomised clinical trials indicate that sitagliptin does not increase cardiovascular risk in patients with T2DM [33]. And chronic treatment with sitagliptin may have cardioprotective effects in diabetes patients presenting with acute coronary syndrome [34].

Furthermore, the anti-atherosclerotic effects of sitagliptin in this study occurred with no differences in food intake, body weight or blood glucose levels between sitagliptin-fed and control animals. Our results are roughly similar to those reported by Junichi Matsubara et al., who reported that sitagliptin reduced atherosclerotic lesion formation in ApoE-deficient mice independent of fasting glucose and lipid profiles [6]. In addition, we unexpected discovered that sitagliptin significantly increase the HDL cholesterol levels and tends to increase the LDL cholestrerol levels in ApoE-/- mice. HDL cholesterol has been demonstrated to reduce the risk of atherosclerosis by multiple pathophysiologic mechanisms [35]. And LDL has been regarded as a positive risk for atherosclerosis when they invade the endothelium and become oxidized. Previous study suggests that sitagliptin is a sound agent for use in the comprehensive treatment of patients with T2DM because it improves not only glycemic control, but also blood pressure and lipid profiles [36]. A systematic review and meta-analysis reported that DPP-4 inhibitors appear to have a beneficial effect on total cholesterol and triglyceride levels, whereas the effect on the other lipids concluded HDL and LDL cholesterol has not been confirmed yet [37]. Whether DPP-4 inhibitor has influence on HDL and LDL cholesterol in mice remains contentious. It has been reported that anagliptin significantly reduced total cholesterol level especially VLDL and LDL without affecting triglyceride level and vildagliptin analogue decreased plasma levels of LDL by 27% in Apoe-/- mice [9,30]. However, some reports showed that sitagliptin has no effect on TG, TC, LDL or HDL levels induced by HFD in Apoe-/- mice [6,10]. Therefore, the definitive effects of DPP-4 inhibitor include sitagliptin on the blood lipid profiles in mice remains contentious.

After observing the anti-atherosclerotic effect of sitagliptin, we then explored its relative mechanisms in further. It has previously been reported that exendin-4, a GLP-1R agonist, attenuates atherosclerosis through PKA–PI3K/Akt–eNOS–p38 MAPK–JNK- dependent pathways via a GLP-1R-dependent mechanism, without affecting metabolic parameters [5,16,18,38-40]. Although studies have shown that sitagliptin can inhibit the formation of atherosclerosis in ApoE-/- mice, the mechanisms through which it attenuates the progress of atherosclerosis are complex and not completely understood.

Recent evidences have shown a promising role for AMPK in the attenuation of atherosclerosis involving vascular dysfunction and endothelial inflammation by upregulating the Akt/eNOS signaling pathway [25,41] and suppressing the activation of ERK1/2 in vascular tissues [24,26]. And it has been demonstrated that mitogen-activated protein kinase (MAPK) may play a role in anti-atherosclerosis [24-29], and moreover alogliptin can inhibit the ERK-mediated expression of matrix metalloproteinases, which are involved in atherosclerosis [17,30]. These informations provided important clues for our study.

Another major finding of this study is that it shows, for the first time, that sitagliptin can activate the AMPK pathway and inhibit MAPK signaling by increasing the phosphorylation of AMPK and its downstream molecule Akt, while reducing the phosphorylation of p38 and ERK1/2 MAPK in the aorta. As a result, sitagliptin reduces serum soluble VCAM-1 and P-selectin levels, which play an important role in regulating the binding of leukocytes to endothelial cells as a key initial step in the formation of atherosclerosis, and also reduces the expression of inflammation factors such as MCP-1 and IL-6. As is well know, inflammation in the vascular endothelium and subsequent leukocyte recruitment are initiating events in the progression of atherosclerosis.

A limitation of the current study is that we have only demonstrated the mechanisms described in aortic tissues, and were unable to provide direct evidence of sitagliptin activating the AMPK and MAPK signaling pathways. Another limitation is that we were unable to measure the plasma levels of active GLP-1 after ingestion. The definitive mechanisms of action for sitagliptin still need further investigation in vitro by culturing primary aortic endothelial cells.

In summary, our study confirms that sitagliptin can attenuate the development of atherosclerosis and alter the composition of the atherosclerotic plaques induced by HFD in ApoE-/- mice. In addition, the beneficial effects of sitagliptin also contain increased HDL cholesterol and decreased adhesion molecules as well as inflammatory cytokines. Our present observations indicate that sitagliptin has protective actions against atherosclerosis via anti-inflammation potentially through AMPK and MAPK-dependent mechanisms. Given that the prevention and treatment of diabetic vascular complications remains important and challenging, sitagliptin, as an effective medicine for diabetes, may open a new therapeutic window for the treatment of atherosclerosis and related diseases.

## Conclusions

Sitagliptin can reduce the atherosclerotic lesion area in ApoE-/- mice by activating AMPK and AMPK-mediated Akt signaling while reducing the phosphorylation of p38 and ERK1/2 MAPK. These, in turn, inhibit inflammatory responses in the aorta, such as the release of MCP-1 and IL-6, and the expression of the adhesion molecules VCAM-1 and serum P-selectin. These effects are independent of weight loss and glucose- and lipid-reducing effects.

## Abbreviations

AMPK: AMP-activated protein kinase; ApoE: Apolipoprotein E null/knockout; cAMP: cyclic AMP; DPP: Dipeptidyl peptidase; eNOS: Endothelial nitric oxide synthase; ERK: Extracellular signal-regulated kinase; GLP: Glucagon-like peptide; GLP-1R: Glucagon-like peptide-1 receptor; HDL: High-density lipoprotein; IL: Interleukin; MAPK: Mitogen-activated protein kinase; MCP: Monocyte chemoattractant protein; NO: Nitric oxide; PCR: Polymerase chain reaction; PI3K: Phosphatidylinositide 3-kinase; PKA: Protein kinase A; VCAM: Vascular cell adhesion molecule.

## Competing interests

The authors declare that they have no competing interests.

## Authors’ contributions

YZ performed the experiments and wrote, reviewed, and edited the manuscript. ZZ and YZ conceived the studies and carried out the molecular biology experiments. JL conducted the animal experiments. LW and FH performed the enzyme-linked immunosorbent assay. WX performed the immunohistochemical experiments. YX performed the sequence alignment. YX, CL and MG were involved in designing and coordinating the study. MG supervised the progress of the experiment and helped to modify the manuscript. All authors read and approved the final manuscript.
